# Development and Validation of a Questionnaire to Measure Serious and Common Quality of Life Issues for Patients Experiencing Small Bowel Obstructions

**DOI:** 10.3390/healthcare2010139

**Published:** 2014-03-07

**Authors:** Amanda D. Rice, Leslie B. Wakefield, Kimberley Patterson, Evette D’Avy Reed, Belinda F. Wurn, Bernhard Klingenberg, C. Richard King, Lawrence J. Wurn

**Affiliations:** 1 Clear Passage Physical Therapy, 4421 NW 39th Ave., Ste 2-2, Gainesville, FL 32606, USA; E-Mails: amandar@clearpassage.com (A.D.R.); lesliew@clearpassage.com (L.B.W.); kimp@clearpassage.com (K.P.); evetter@clearpassage.com (E.D.R.); belindaw@clearpassage.com (B.F.W.); richardk@clearpassage.com (C.R.K.); 2 Department of Mathematics and Statistics, Williams College, Williamstown, MA 01267, USA; E-Mail: bernhard.kingenberg@williams.edu

**Keywords:** small bowel obstruction, quality of life questionnaire, validated survey, small bowel obstruction questionnaire

## Abstract

A validated questionnaire to assess the impact of small bowel obstructions (SBO) on patients’ quality of life was developed and validated. The questionnaire included measurements for the impact on the patients’ quality of life in respect to diet, pain, gastrointestinal symptoms and daily life. The questionnaire was validated using 149 normal subjects. Chronbach alpha was 0.86. Test retest reliability was evaluated with 72 normal subjects, the correlation coefficient was 0.93. Discriminate validity was determined to be significant using the normal subject questionnaires and 10 questionnaires from subjects with recurrent SBO. Normative and level of impact for each measured domain were established using one standard deviation from the mean in the normal population and clinical relevance. This questionnaire is a valid and reliable instrument to measure the impact of SBO on a patient’s quality of life related to recurrent SBOs; therefore establishing a mechanism to monitor and quantify changes in quality of life over time.

## 1. Introduction

Small bowel obstruction (SBO) is a common medical occurrence, most commonly a complication of surgery secondary to the formation of post surgical adhesions [1,2]. It has been reported previously that 50%–93% of patients that undergo abdominopelvic surgery will develop adhesions [3,4,5,6] and 35% will be hospitalized for a bowel obstruction within 10 years of the first surgery [1,2,5,6,7,8,9]. The diagnosis of SBO is based upon symptomatic complaints and radiological tests, with resolution of the SBO determined by improvement in radiological tests and/or resolution of patient reported symptoms in cases of nonsurgically treated obstructions [9,10]. A wide variety of symptoms accompany a bowel obstruction, impacting multiple aspects of life. Patients with recurrent obstructions often have a limited or liquid diet requirement, experience pain in multiple areas of their bodies, and have gastrointestinal symptoms that impact their quality of life including bloating, nausea, vomiting, diarrhea or constipation, and the inability to plan or engage in normal social activities.

Currently, there is no validated tool to assess patient reported quality of life specific to patients experiencing SBOs. SBOs and the experiences of the patients with recurrent SBOs are not directly comparable to other gastrointestinal problems for which validated questionnaires are available. The questionnaire for patients with functional gastrointestinal disorders does not address pain or diet [11,12]; the QOL questionnaire for patients with a colostomy or ileostomy is not generally applicable to the SBO population [13,14]; the bowel function index is not complete and focused on opioid induced constipation [15]; and the health related quality of life (HRQOL) measurement questionnaires are general and do not address the specific symptoms experienced by SBO patients [13,14,16,17]. Furthermore, clinicians rely upon subjective, qualitative measures reported by patients presenting with symptoms of SBO. A quantitative noninvasive tool capable of being used in both acute presentation of SBO and also in routine care settings to track the symptoms and impact on QOL could allow for earlier interventions and can aid in measurement of treatment regimen efficacy for patients.

Therefore, we developed a questionnaire to provide an instrument to measure various aspects of life impacted by SBO specific to this patient population. This instrument was designed to assess the degree to which a patient’s quality of life is affected by the symptoms of recurrent SBOs including the overall domains of diet, gastrointestinal (GI) symptoms, pain, quality of life (QOL) and medication requirements.

## 2. Experimental

### 2.1. Instrument Development

The questionnaire was developed by an independent physical therapy group that included treating therapists, the clinic’s medical director, biostatisticians and collaborative scientists to address areas of life impacted by small bowel obstructions. Development was based on standard clinical observations and patient responses over the course of 4 years from intensive interviews with patients whose lives were disrupted by bowel obstructions. We noted histories, symptoms, pain, diet, concerns for the future, and other conditions that patients reported as negatively affecting their quality of life. This instrument was designed to measure improvement of self reported symptoms related to SBOs after physical therapy treatment for abdominal adhesions. A total of 48 English speaking patients provided feedback during the development of the instrument, 34 female and 14 male with an average age of 53.9 ± 16 years. Each of the patients had experienced recurrent SBOs, ranging from two episodes over their lifetime to monthly episodes of partial SBOs. During the initial interviews with the physical therapists that lasted on average 60 min, patients were queried regarding symptomatic complaints that were current as well as those experienced during a partial SBO episode. The physical therapists noted the symptoms that caused the patients the highest degree of stress and negative impact upon their life. Based upon the symptomatic complaints from these 48 patients and scientific input, the questionnaire was developed to assess the most common symptomatic complaints of this patient population. The questions were designed to be easy to understand and administer, to provide an overall quantitative score for the impact on quality of life due to SBO, and to provide an unbiased report of QOL irrespective of age, level of education, gender or economic status.

A total of 38 questions were used in the final questionnaire. Questions were divided into domains of diet, GI symptoms, pain, medication requirement, and overall QOL, with additional pain rating sections utilizing the standard pain scale as well as duration of pain. There were five possible responses for the diet, GI symptom, pain, medication and QOL questions, which were based upon the number of days over the last month that the subject experienced the symptom described in the question. Positive associated experiences were scored from 4 to 0 with 4 being experienced virtually every day (28–30 days over the last month) and 0 being experienced never or almost never (fewer than 3 days over the last month). Negative associated experiences were scored from 0 (experienced virtually every day) to 4 (experienced never or almost never). The score for pain duration was rated from 0 (no pain) to 5 (pain lasting longer than 3 h) and values for the level of pain experienced were rated from 0 to 10 using the standard pain scale. The survey and scoring grid is located in Tables S1–S3. 

### 2.2. Data Collection

Data were collected from two distinct populations: patients with a history of SBO in a focus group, and a population of normal subjects obtained using the online service Survey Monkey (Portland, OR, USA). The questionnaire was formatted for use on Survey Monkey to establish values for each question and domain for a normal population. Subjects with a history of abdominal or pelvic surgery, bowel obstructions, radiation or chemotherapy were excluded from the study. The SBO questionnaire was included in its entirety with the addition of questions for demographics (gender, education, household income, marital status) and one quality assurance question in which the respondent was instructed to select a specific answer. Subjects were between the ages of 18–65 and no identifying personal information was collected from subjects in the Survey Monkey arm of the study.

A subset of subjects was re-contacted by Survey Monkey to complete the survey again 44–46 days after completing the survey the first time to allow for assessment of test-retest validity for the survey. It was assumed there would be no variation in symptoms over the 6 week time frame. Validation of the questionnaire in subjects with a history of recurrent SBOs was performed using a focus group of 10 patients. These patients were treated for abdominal adhesions in the clinic and had a history of SBO. MaGil Institutional Review Board approved this study.

### 2.3. Statistics

Descriptive statistics were calculated for all domains for both the normal population and SBO groups. Two tailed, unequal variance T-tests were performed for each domain for comparison of the means for the normal population and SBO patient population. Bonferroni multiplicity adjusted *p* values were determined using standard methods. All statistical analyses were completed using standard protocols in Microsoft Excel.

## 3. Results and Discussion

### 3.1. Study Population

The normal population included 258 subject-attempted questionnaires with 149 included in the final analysis, completed via Survey Monkey. Questionnaires in which the subject responded “yes” to questions regarding previous abdominal surgery (69 questionnaires) or previous SBO (7 questionnaires) were excluded from the analysis. An additional 62 questionnaires were excluded for either an incorrect answer to the quality control question or failure to answer 95% or more of the questions. A total of 234 questionnaires were kept for the final analysis, including both initial and retest questionnaires. A total of 10 SBO focus group patient questionnaires were included in the analysis. The sociodemographics of the subjects are located in Table 1 and Table 2. While the SBO population was mainly Caucasian male, the normal population group had a wide variation in all demographics and included 51% female subjects.

### 3.2. Validation Study

The primary analysis for reliability of the questionnaire in a normal population included 149 normal subject questionnaires for determining reliability and Chronbach’s α. There were 72 repeat questionnaires which were compared to the first questionnaires values and retest validity determined by Pearson product-moment correlation. The normal population general statistics are located in Table 3 for each domain measured.

### 3.3. Reliability

The Chronbach α coefficient to assess internal consistency for the questionnaire overall in a normal population was 0.86. The split half coefficient was 0.88 for the normal population. The questionnaire had similar values for Chronbach α and split half for the SBO patient population with values of 0.86 and 0.94, respectively. This demonstrates a reliable tool over two different populations, where values for Chronbach α over 0.7 and split half of 0.6 are the minimum acceptable values.

Chronbach α and split half were calculated for each group after conversion to domain scores. The normal population demonstrated good internal consistency with values of 0.77 for Chronbach α and 0.73 for split half; the SBO patient population also demonstrated acceptable internal consistency with values of 0.81 for Chronbach α and 0.99 for split half analysis.

**Table 1:** Demographics of normal population subjects included in this study.

Variable	Answer	*N*	%
**Gender**	Male	72	48.3
	Female	76	51.0
	Not answered	1	0.7
**Race/Ethnicity**	White	129	86.6
	African American or black	11	7.4
	Asian	4	2.7
	Multiple Races	3	2.0
	Other	1	0.7
	Not answered	1	0.7
**Marital Status**	Married/Long-term relationship	99	66.4
	Single	28	18.8
	Divorced/widowed	21	14.1
	Not answered	1	0.7
**Education**	Did not complete high school	2	1.3
	High School Graduate/GED	39	26.2
	1–3 years college	46	30.9
	4 years college	34	22.8
	Some graduate school	5	3.4
	Completed graduate school	21	14.1
	Not answered	2	1.3
**Household Income**	$0–$24,999	27	18.1
	$25,000–$49,999	36	24.2
	$50,000–$74,999	33	22.1
	$75,000–$99,999	23	15.4
	over $100,000	29	19.5
	Refused to answer	1	0.7

**Table 2:** Demographics for small bowel obstruction (SBO) patients included in this study.

Variable	Answer	*N*	%
**Gender**	Male	7	70.0
	Female	3	30.0
**Race/Ethnicity**	White	7	70.0
	African American or black	2	20.0
	Asian	1	10.0
**Marital Status**	Married/Long-term relationship	7	70.0
	Single	3	30.0
**Education**	Did not complete high school	0	0.0
	High School Graduate/GED	0	0.0
	1–3 years college	0	0.0
	4 years college	2	20.0
	Some graduate school	0	0.0
	Completed graduate school	1	10.0
	Not answered	6	60.0

**Table 3:** Normal population basics statistics for the domains in the SBO QOL questionnaire. CI = confidence interval.

Domain	Mean	Median	One Standard Deviation	Two Standard Deviation	−95%CI	+95%CI	Score Range
Diet	15.1342	16	1.9475	3.8950	14.8215	15.4469	0 to 16
Pain	4.8792	4	5.2547	10.5094	3.9968	5.7616	0 to 40
Medication	0.6779	0	1.0583	2.1165	0.4592	0.8965	0 to 4
GI Symptoms	3.0134	2	3.0388	6.0775	2.4031	3.6237	0 to 44
QOL	0.5772	0	1.0456	2.0911	0.3462	0.8082	0 to 28
Average Pain Level	1.2617	0	1.7972	3.5944	0.9069	1.6166	0 to 10
Maximum Pain Level	1.5772	0	2.3986	4.7973	1.1577	1.9967	0 to 10
Minimum Pain Level	0.9396	0	1.5185	3.0371	0.6255	1.2537	0 to 10
Duration of Pain	1.2282	0	1.4743	2.9485	0.9935	1.4629	0 to 5

The test-retest reliability was determined by calculation of Pearson’s correlations for all normal subjects that completed the questionnaire twice using the SurveyMonkey platform. The correlation for the entire questionnaire was 0.93; and 0.93 for the calculated domains for this group of 72 normal subjects, demonstrating good reliability for this instrument and the established domains.

### 3.4. Validity

**Figure 1:**
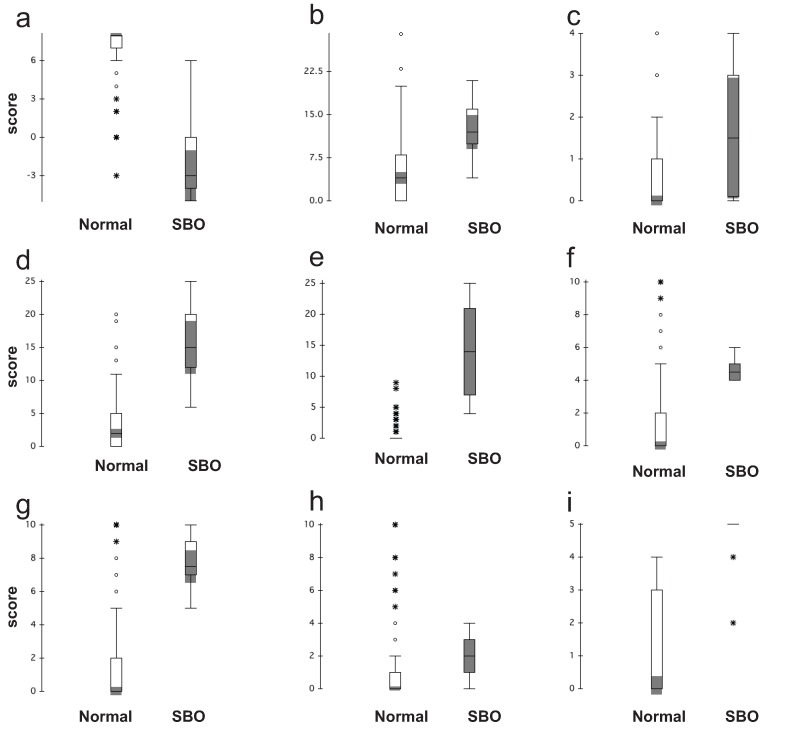
Box and whisker plots for normal and SBO populations for each domain in the SBO survey. Each domain is represented separately: (**a**) diet; (**b**) pain; (**c**) medication; (**d**) gastrointestinal symptom; (**e**) quality of life; (**f**) average pain on the pain scale; (**g**) maximum level of pain on the pain scale; (**h**) minimum level of pain on the pain scale; and (**i**) duration of pain. The box represents the 25% to 75% percentile values; the horizontal line is the median. The whiskers on the box show the main data while extreme outliers are denoted by open circles and very extreme outliers are denoted by a starburst. The shading shows the 95% confidence interval around the median.

Discriminate validity was assessed by comparing the calculated sum-mean values for each of the domains for the normal and SBO patient populations to demonstrate that each population was distinct. Box and whisker plots showing the ranges for responses in each group are located in Figure 1. There were clear differences between the two groups in all domains except pain and medication, which was to be expected, based upon published studies outlying the large percentage of the normal population with chronic pain [18,19,20].

Table 4 shows multiplicity adjusted *p*-values for comparing mean scores between the normal and SBO population, across all domains. All the cumulative quality of life domains (diet, pain, GI symptoms and QOL) showed a significant difference. Single question domains (medication, pain duration and pain scores) were more variable, however significant differences were observed in the duration and level of pain reported by the subjects with the SBO population reporting more severe, sustained pain.

### 3.5. Establishment of Normative Values for Domains

Normative ranges for each domain were determined based upon the 25–50–75 percentiles for each domain score ranges, while ensuring clinical relevance (See Table 5). The prescribed ranges were supported in the standard deviation for the respective domains in the normal subject population (Table 3). There are four distinct ranges for each domain: no impact, mild impact, moderate impact, and severe impact shown in Table 6. The normative values for medication usage were assigned based upon clinical relevance; where it was determined to be clinically significant for any subject to require medication more than 10 days a month for this instrument.

The ranges for pain duration and intensity are not cumulative and are represented in this questionnaire as single items to assess the change of pain experienced over time, in line with other validated questionnaires in use for various other conditions. No single range was identified as “normal” or “no impact” due to the variability in these measures that have been reported by others and the overall design for this questionnaire to be used as a tool to monitor change and/or improvements with treatment. A significant improvement or change in reported pain scores is accepted as a 2 point change per the FDA guidelines and large published studies, therefore a change in the visual analog pain scale of 2 points or greater is considered a significant change [21,22].

Based upon our assigned ranges for all the domains, it was observed that 89.3% of the normal population fell within the defined no impact category for diet while only 10% of SBO subjects reported no impact in their diet. Within the pain domain, 83.2% of normal subjects reported composite scores within the no impact range, compared to 30% of the SBO subjects. The normal population scores fell within the no impact range for QOL and GI symptoms 98.6% and 96.6% respectively; the SBO subjects’ scores for no impact QOL was 30% and 20% for GI symptoms. The definition of a normal population was quite broad for this study, excluding only subjects with a history of bowel obstruction, cancer, chemotherapy, radiation or surgery. Given these exclusionary criteria, it was expected that 10%–30% of the respondents would have a chronic pain, gastrointestinal disease or other contributory condition shifting their composite score for a domain outside of the no impact range that would be expected in a normal population [17,18,19,20,23].

**Table 4:** Comparison of the normal population and SBO groups for condition specific validity for all domains measured in the SBO QOL questionnaire.

Group	Domain Mean(SD) Scores	T-Test	Bonferroni Adjusted
**Diet**			
SBO	5.4(4.3)	<0.0001	<0.0009
Normal	15.13(1.9)		
**Pain**			
SBO	12.4(4.7)	0.0006	0.0052
Normal	4.88(5.5)		
**Medication**			
SBO	1.7(1.49)	0.0615	0.5533
Normal	0.68(1.4)		
**GI Symptoms**			
SBO	15.4(6.4)	0.0002	0.0014
Normal	3.01(3.8)		
**QOL**			
SBO	14.3(7.7)	0.0003	0.0029
Normal	0.58(1.43)		
**Average Pain Level**			
SBO	4.7(0.8)	<0.0001	<0.0009
Normal	1.26(2.21)		
**Maximum Pain Level**			
SBO	7.8(1.7)	<0.0001	<0.0009
Normal	1.6(2.6)		
**Minimum Pain Level**			
SBO	1.9(1.2)	0.0369	0.3320
Normal	0.94(1.96)		
**Duration of Pain**			
SBO	4.6(0.97)	<0.0001	<0.0009
Normal	1.2(1.5)		

**Table 5:** Domain quartiles for each measured domain in the SBO QOL questionnaire based upon one standard deviation in a normal population.

Domain	Minimum	25%	50%	75%	Maximum
Diet	0	4	8	12	16
Pain	0	10	20	30	40
Medication	0	1	2	3	4
GI Symptoms	0	11	22	33	44
QOL	0	7	14	21	28

**Table 6:** Grouping of degree of impact on quality of life for each measured domain. The sum of the domain scores is used to determine the classification in the degree of impact on the patient’s quality of life for that domain. No impact is defined for this questionnaire as the expected values in a normal healthy individual.

Domain	No Impact	Slight Impact	Moderate Impact	Severe Impact
Diet	13–16	9–12	5–8	0–4
Pain	0–9	10–19	20–29	30–40
GI symptoms	0–10	11–21	22–32	33 to 44
QOL	0–7	8–14	15–21	22–28
Medication	0–1	2	3	4

## 4. Conclusions

To the knowledge of the authors, there was not a questionnaire specific for patients with SBO to quantify the impact of SBO on their overall quality of life prior to this study. This validated questionnaire allows for unbiased, qualitative assessment of changes in domains of life impacted by SBO symptoms that has been demonstrated to have good reliability and validity via Chronbach, split half and Pearson analysis. Furthermore, this questionnaire provides a tool for clinical trials and use in general practice that is noninvasive and patient-centered to monitor changes over time in the various domains of life impacted by SBOs.

SBO is a complex condition, impacting not only patients’ gastrointestinal tract and pain levels, but also their ability to perform normal life tasks. A validated QOL questionnaire is needed given that a large proportion of the population have had or will have a non-malignant bowel obstruction secondary to abdominal or pelvic surgery [2,7,24,25,26]. This non-invasive, quantitative, self reporting instrument to determine the impact of the SBO symptoms on a patient’s quality of life is useful to help examine disease progression, overall treatment efficacy, and to aid in decisions for treatment.

The limitations of this study include a small number of SBO subject responses in the focus group, however the discriminate validity was significant; therefore this is believed to not be an issue. Both genders were combined for this analysis and establishment of normative values for each of the domains; however, this is justified as there were no large variations between genders. In addition, the definition of a normal population for the purpose of the SurveyMonkey subject recruitment was not overly stringent in excluding those subjects with chronic pain or functional GI disorders. This was justified in the fact that these subjects are a part of the normal population and did not have a history of previous SBO.

## Supplementary Files

Supplementary File 1
