# Supplementary material for: Development and Validation of a Questionnaire to Measure Serious and Common Quality of Life Issues for Patients Experiencing Small Bowel Obstructions
Source: Healthcare (Basel). 2014 Mar 7;2(1):139–49. doi: 10.3390/healthcare2010139 (PMC4934500; doi:10.3390/healthcare2010139)
Supplement: Supplementary File 1 [file healthcare-02-00139-s001.pdf]

Supplementary Information

Table S1. Small Bowel Obstruction Questionnaire. The point value for each question is denoted in the boxes. Instructions: These questions ask about your diet, digestion and pain related to your bowels during the past 30 days. Please select the best answer for each question. Begin each question with “During the last 30 days”.

#	Question	**Virtually Every Day** 28–30 days	**Most Days** 20–28 days	**About Half of the Days** 11–19 days	**Some Days** 3–10 days	**Never or Almost never** Less than 3 days
	**Diet**					
1	I was on a totally liquid diet	0	1	2	3	4
2	I was on a soft food diet	0	1	2	3	4
3	I could easily ingest and digest solid food	4	3	2	1	0
4	I could eat whatever I liked, without problems	4	3	2	1	0
	**Pain**					
5	I had pain	4	3	2	1	0
6	I had pain at or above my belly button	4	3	2	1	0
7	I had pain below my belly button	4	3	2	1	0
8	I had pain with bowel movements	4	3	2	1	0
9	I experienced head or neck pain	4	3	2	1	0
10	I had Migraine headache(s)	4	3	2	1	0
11	I experienced tailbone (coccyx) pain	4	3	2	1	0
12	Eating caused my abdomen to hurt	4	3	2	1	0
13	Drinking liquids caused my abdomen to hurt	4	3	2	1	0
14	I had back pain	4	3	2	1	0
	**Other Adverse Experiences**					
15	I had nausea after eating	4	3	2	1	0
16	I vomited after eating	4	3	2	1	0
17	I experienced digestive spasm	4	3	2	1	0
18	I had constipation	4	3	2	1	0
19	I had diarrhea	4	3	2	1	0
20	I eliminated blood-stained, or jelly-like mucus	4	3	2	1	0

Table S1. *Cont.*

#	Question	**Virtually Every Day** 28–30 days	**Most Days** 20–28 days	**About Half of the Days** 11–19 days	**Some Days** 3–10 days	**Never or Almost Never** Less than 3 days
21	I had gas/bloating/distension	4	3	2	1	0
22	I had increased bowel sounds	4	3	2	1	0
23	I was unable to have bowel movements when I wanted or needed to go	4	3	2	1	0
24	My bowel movements looked abnormal	4	3	2	1	0
25	Eating or drinking caused me to swell, bloat or have gas	4	3	2	1	0
**Daily Life**						
26	I took medications for my symptoms	4	3	2	1	0
27	I was unable to work due to my condition	4	3	2	1	0
28	I was unable to have a normal social life due to my condition	4	3	2	1	0
29	I would feel uncomfortable or unable to make travel plans due to my condition	4	3	2	1	0
30	My sex life suffered due to my condition	4	3	2	1	0
31	I had decreased ability to participate in normal daily activities due to my condition	4	3	2	1	0
32	I would be reluctant to eat at a restaurant or a friend's house due to my condition	4	3	2	1	0
33	I felt that I could decrease my own symptoms using my hands	0	1	2	3	4
34	I worried about having another bowel obstruction	4	3	2	1	0

Table S2. The longest duration of my worst pain was (mark one answer by placing an X).

Longer than Three Hours	Up to Three Hours	Ten Minutes to an Hour	One to Ten Minutes	Only a Few Seconds	I did not Have Pain
5	4	3	2	1	0

Table S3. With zero being no pain and ten being the worst pain I could imagine, the pain levels in or from my bowels over the last four weeks were (mark one in each row by placing an X).

Pain Level	0	1	2	3	4	5	6	7	8	9	10
**Highest** Pain:											
**Lowest** Pain:											
**Average** Pain:											

Scoring:

Domain	Sum of Scores for Questions
**Diet**	1–4
**Pain**	5–14
**Gastrointestinal Symptoms**	15–25
**Quality of Life**	27–34
**Medication**	26
**Pain Duration**	35
**Pain Characteristics (Highest, Lowest, Average)**	36

Quartiles by Domain Score

Normative Values per Domain				
**Domain**	**Classification from Sum of Scores**			
	**Normal**	**Slight Impact**	**Moderate Impact**	**Severe Impact**
**Diet**	13–16	9–12	5–8	0–4
**Pain**	0–9	10–19	20–29	30–40
**Gastrointestinal Symptoms**	0–10	11–21	22–32	33–44
**Quality of Life**	0–7	8–14	15–21	22–28
**Medication**	0–1	2	3	4

© 2014 by the authors; licensee MDPI, Basel, Switzerland. This article is an open access article distributed under the terms and conditions of the Creative Commons Attribution license (http://creativecommons.org/licenses/by/3.0/).
